# Anti-claudin 18.2 antibody as new targeted therapy for advanced gastric cancer

**DOI:** 10.1186/s13045-017-0473-4

**Published:** 2017-05-12

**Authors:** Prabhsimranjot Singh, Sudhamshi Toom, Yiwu Huang

**Affiliations:** 1 Hematology/Oncology Fellowship Program, Maimonides Medical Center, Brooklyn, NY USA; 2 Department of Internal Medicine, Maimonides Medical Center, Brooklyn, NY USA; 3 Division of Hematology/Oncology, Maimonides Medical Center, Brooklyn, NY USA

**Keywords:** Gastric cancer, Claudiximab, IMAB362, Targeted therapy, Anti-claudin antibody

## Abstract

Targeted therapy and immunotherapy have revolutionized treatment of various cancers in the past decade. Despite targeted therapy with trastuzumab in Her2-positive gastric cancer patients, survival has been dismal, mostly due to disease progression and toxicity related to the treatments. One area of active development is looking for ideal monoclonal antibodies (IMAB) specific to the proteins only on the tumor and hence avoiding unnecessary side effects. Claudin proteins with isoform 2 are one such protein, specific for several cancers, particularly gastric cancer and its metastases, leading to the development of anti-claudin 18.2 specific antibody, claudiximab. This review will highlight the latest development of claudiximab as first in class IMAB for the treatment of gastric cancer.

## Background

Gastric cancer is one of the most common cancers worldwide, the fourth (in males) and fifth (in females) most common causes of cancer-related deaths in the developed world. An estimated 951,600 new stomach cancer cases and 723,100 deaths occurred in 2012. The incidence of gastric cancer varies widely according to geographic region [1]. The majority of patients with gastric cancer are often diagnosed in the advanced stage of the disease. Early stages of gastric cancer are potentially curable with radical gastrectomy, although approximately 50% recur [2]. Adjuvant chemotherapy and chemo-radiation have led to improvement in overall survival (OS) [3]. Advanced gastric cancer is not curable and treatment currently is palliative chemotherapy conferring a median survival time of 8–10 months [4]. Multiple new chemotherapy regimens are studied with improved response rates and tolerability; however, the 5-year survival rates are dismal.

Immunotherapy and targeted agents like trastuzumab, ramucirumab, and tyrosine kinase inhibitors have revolutionized the treatments of various cancer including gastric cancer [5–16]. With the advent of targeted therapy, different molecules targeting different pathways were developed for the treatment of gastric cancer. In this review, we outline recently developed molecularly targeted therapy against claudin receptors—claudiximab (previously IMAB362). Claudiximab is first-in-class chimeric monoclonal antibody-IMAB (ideal monoclonal antibody), for the treatment of gastric cancer. IMABs bind to cancer-selective targets that are predominantly expressed in tumor cells and show little or no expression in healthy tissues. This unique cancer-cell selectivity of IMABs allows for maximal anticancer potency while diminishing toxicity. They have broader therapeutic window allowing optimal dosing.

## Claudin proteins

**Fig. 1:**
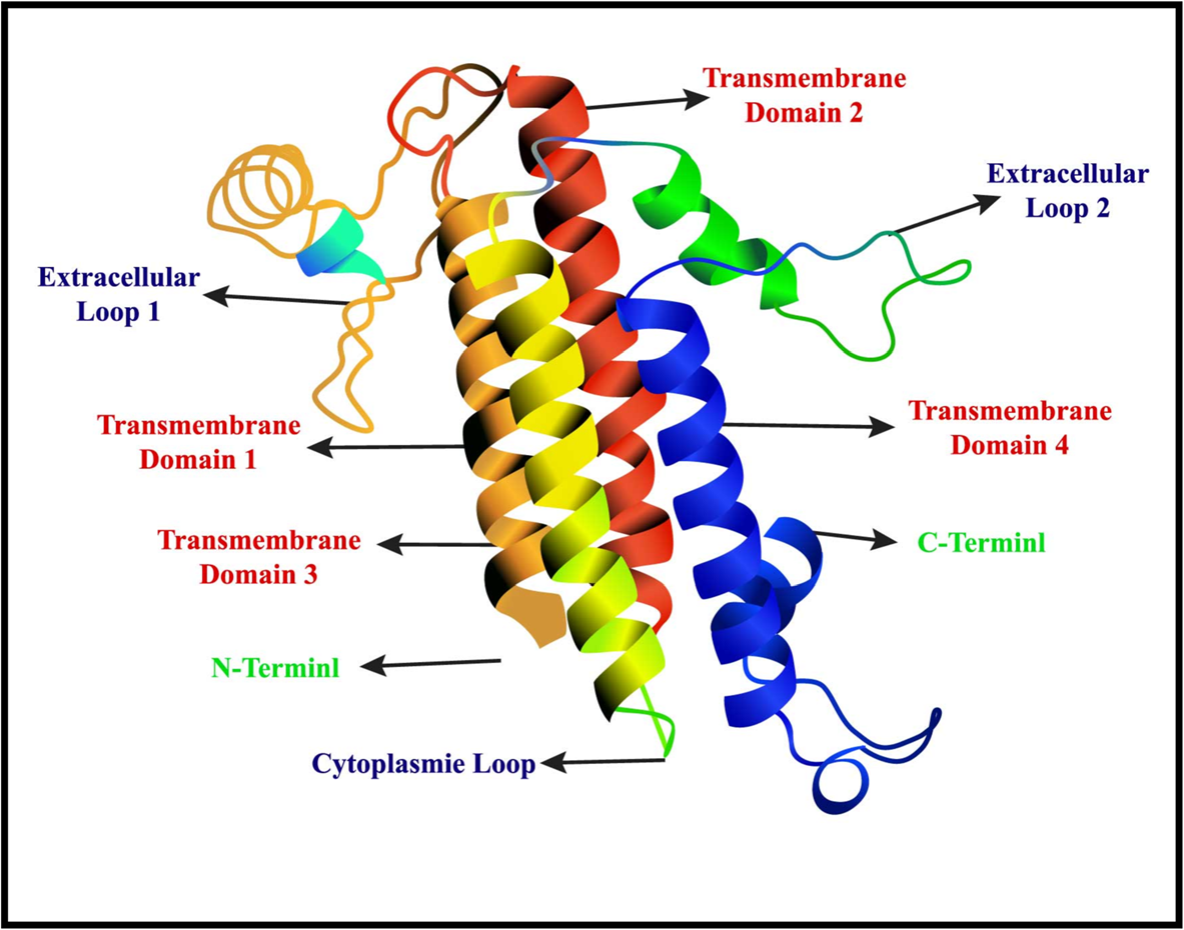
Model structure of claudin protein

Claudins are a family of proteins, first described by Shorichiro Tsukita et al. in 1998 which form the important components of the tight cell junctions [17]. They establish a paracellular barrier which controls the flow of molecules between the cells. The transmembrane domains of claudins include a N-terminus and a C-terminus in the cytoplasm (Fig. 1). Different claudins are expressed on different tissues, their altered function has linked to formation of cancers of respective tissues [18, 19]. Claudin-1 expression has been shown to have prognostic value in colon cancer [20], claudin-18 in gastric cancer [21], and claudin-10 in hepatocellular carcinoma [22]. Claudins, being surface proteins, represent a useful target for various therapeutic strategies.

Ugur Sahin et al. identified isoform 2 of the tight junction molecule claudin-18 (CLDN18.2) as a highly selective cell lineage marker [23].They observed its expression in normal tissues is strictly confined to differentiated epithelial cells of the gastric mucosa, but it was absent from the gastric stem cell zone. Claudin 18.2 was retained on malignant transformation and was expressed in a significant proportion of primary gastric cancers and its metastases. Frequently ectopic activation of claudin 18.2 was also found in pancreatic, esophageal, ovarian, and lung tumors. The study suggested that CLDN18.2 has highly restricted expression pattern in normal tissues, with frequent ectopic activation in a diversity of human cancers.

Claudin 18.2 is involved in tumor development and progression and located in the outer cell membrane. It has exposed extracellular loops and is available for monoclonal antibody binding. These biological characteristics suggested that it is an ideal molecule for targeted therapy and led to the further development of monoclonal antibodies against claudin 18.2, such as claudiximab (IMAB362).

## Claudiximab (IMAB362)

**Fig. 2:**
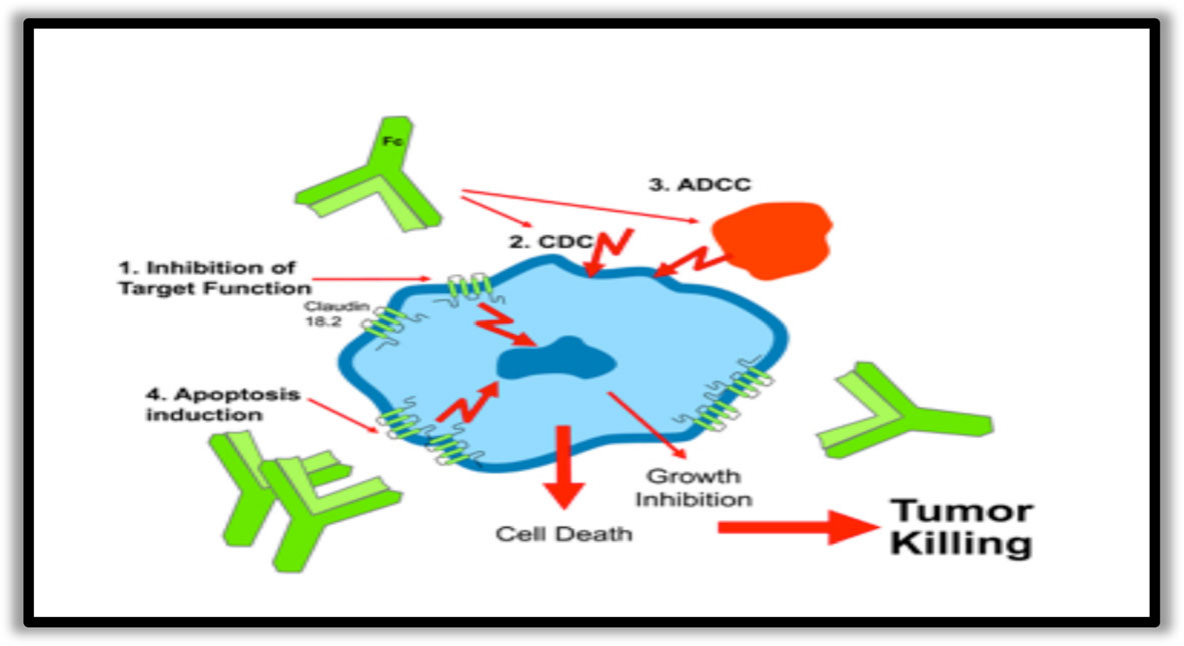
Mechanism of action of claudiximab (IMAB362)

Claudiximab is a novel chimeric IgG1 antibody highly specific for claudin 18.2. Claudiximab is derived from a murine monoclonal antibody and has been chimerized to display the human IgG1 constant region for clinical use. Claudiximab is produced in Chinese Hamster Ovary (CHO) cells by standard recombinant expression technology. It binds to claudin 18.2 on the tumor cell surface to stimulate cellular and soluble immune effectors that activate antibody-dependent cytotoxicity (ADCC) and complement dependent cytotoxicity (CDC). It can also induce apoptosis and inhibit cell proliferation. When combined with chemotherapy, claudiximab enhances T-cell infiltration and induce pro-inflammatory cytokines (Fig. 2).

The safety of the claudiximab was extensively assessed in preclinical animal models. In preclinical studies, claudiximab proved to inhibit tumor growth and eradicate cancer cells by mechanisms such as complement-dependent cytotoxicity (CDC) and antibody-dependent cellular cytotoxicity (ADCC) [24].

## Claudiximab for advanced gastric cancer treatment in clinical trials

Currently, claudiximab is studied in numerous clinical trials for the treatment of patients with advanced gastroesophageal cancer. Data reported in the clinical trials of claudiximab are summarized in Table 1.

**Table 1:** Various clinical trials involving claudiximab (IMAB362)

Study-completion date	NCT number	Phase	Number of patients	Design	Response rate	OS	Median FPS	Adverse events
**–**	NCT00909025	I	15	Single-dose escalation safety study	**–**	**–**	**–**	**–**
PILOT-2014	NCT01671774	I	20	Multiple dose, immunomodulation IMAB362 + zoledronic acid + IL-2	11 patients had disease control	40 weeks	12.7 weeks	Nausea and vomiting
MONO-2013	NCT01197885	IIA	54	Single arm, repeated dose, monotherapy study	Response rate-10% Disease control rate-30%	**–**	14.5 weeks	Vomiting
FAST-2015	NCT01630083	IIB	161 +85	Randomized EOX vs claudiximab + EOX, extended with high-dose claudiximab + EOX	Objective response rate- 25 vs 39%	8.4 vs 13.4 months	4.8 vs 7.9 months	Vomiting, neutrope-nia, and anemia

The first in-human phase I single-dose escalation study enrolled 15 patients with treatment-refractory metastatic gastroesophageal adenocarcinomas with CLDN18.2-positive tumors as determined by CLAUDETECT™ 18.2 Kit by immunohistochemistry test [24]. Patients were enrolled into five dose groups from 33 to 1000 mg/m^2^ with three patients in each group. Patients were followed up for 4 weeks. If disease control was achieved, claudiximab was allowed to be continued until progression. Phase I trial reported all dose levels of claudiximab were generally well tolerated with nausea, and vomiting were the most common adverse events (AEs). No dose-limiting toxicity was observed at single doses up to 1000 mg/m^2^. Based on pharmacokinetic considerations and preclinical dose/response data, a 600 mg/m^2^ dose was recommended for the phase II multidose trial (NCT00909025).

In another phase I trial “PILOT” evaluated the safety and efficacy of claudiximab in combination with zoledronic acid (ZA) and interleukin-2(IL2) in patients with CLDN18.2+ gastroesophageal adenocarcinomas [25]. Twenty-eight patients who had progressed on more than one chemotherapy regimen were enrolled and received claudiximab (800 mg/m2 in cycle [cy] 1, followed by 600 mg/m2 in subsequent cy, IV q3w) plus ZA (4 mg, IV, day 1 of cy 1 and 3) in arm 1; claudiximab, ZA plus low IL-2 (1 × 10^6^ IU, s.c., days 1–3 of cy 1 and 3) in arm 2; claudiximab, ZA plus intermediate IL-2 (3 × 10^6^ IU s.c., days 1–3 of cy 1 and 3) in arm 3; and IMAB362 alone in arm 4. Of the 20 evaluable patients, 11 patients achieved disease control, 1 patient had unconfirmed partial response; the rest 10 patients had stable disease. The median progression-free survival was 12.7 weeks, and median overall survival was 40 weeks. Claudiximab alone and in combination was well tolerated and common treatment-related adverse events (AEs) included nausea and vomiting mostly grades 1–3. The study concluded that claudiximab has anti-tumor activity as monotherapy and can also be safely combined with ZA/IL2 in the treatment of advanced gastroesophageal cancers (NCT01671774).

This was followed by a phase IIa (MONO) study aimed at establishing the efficacy and safety of multiple doses of claudiximab as monotherapy in patients with metastatic, refractory, or recurrent adenocarcinoma of the stomach or the lower esophagus [26]. The study enrolled 54 patients; with four patients receiving biweekly 300 mg/m^2^ and 50 receiving 600 mg/m^2^ biweekly as 2 hour infusion. Patients with disease control (*N* = 9) were allowed to continue claudiximab therapy until progression. Forty patients all in the 600 mg/m^2^ were evaluable for analysis. The response rate was 10%, and the disease control rate was 30% (best observed response: PR, *n* = 4 and SD, *n* = 8). Median PFS was 102 days (95% CI, 70–146 days). All observed AEs were grades 1–3. The most frequent grade 3 AEs were vomiting in 31 pts. No grade 4 AEs occurred. Pharmacokinetics from the study supported 3-weekly IV dosing (NCT01197885).

Subsequently, a phase IIb (FAST) study evaluated claudiximab as first line in patients with advanced/recurrent gastric and GEJ cancer. Patients with CLDN18.2 expression of ≥2+ in ≥40% tumor cells (as validated by CLAUDETECT™ 18.2 Kit), Eastern Cooperative Oncology Group performance status of 0–1 and who were not eligible for trastuzumab were included in the study [27, 28]. Seven hundred thirty-nine patients were consented and 352 (48%) were tested CLDN18.2+ per protocol criteria. Of those, 161 patients (gastric, 80%; GEJ, 16%; esophageal, 4%) were randomized in 1:1 to first-line EOX (epirubicin 50 mg/m2, oxaliplatin 130 mg/m2 d1, and capecitabine 625 mg/m2 bid, d1–21, every 21 days) with or without claudiximab (loading dose 800 mg/m2, then 600 mg/m2 d1, every 21 days). The study had an exploratory arm three (*N* = 85) to investigate a high-dose claudiximab (1000 mg/m2) plus EOX. The study met its primary end point of progression-free survival (PFS). Claudiximab plus EOX significantly improved PFS (median 7.9 vs 4.8 months; HR 0.47; *p* = 0.0001) and OS (median 13.3 vs 8.4 months; HR 0.51, *p* < 0.001) compared to EOX alone. Subgroup analysis of patients with very high CLDN18.2 expression (≥2+ intensity in ≥70% tumor cells), outcomes were more pronounced (PFS, 7.2 vs 5.6 months; HR 0.36; *p* = 0.0005; OS, 9.0 vs 16.7 months; HR 0.45, *p* < 0.0005). Patients who received claudiximab also showed a higher objective response rate (ORR) at 39% compared with 25% in the EOX arm. In the claudiximab cohort, 8 patients (10.4%) achieved a complete response (CR), 22 patients (28.6%) had a partial response (PR), and 34 patients (44.2%) had stable disease (SD). With chemotherapy, 3 patients (3.6%) achieved a CR, 18 (21.4%) had a PR, and 43 (51.2%) had SD. Disease progression was experienced by 5.2 and 11.9% of patients receiving claudiximab vs chemotherapy, respectively. The treatment was well tolerated with mostly grade 1/2-related adverse events which included vomiting, neutropenia, and anemia. Grade 3/4 events were not significantly increased in patients receiving claudiximab. Overall, 55.8% of patients in the investigational cohort showed grades 1/2 vomiting, and 10.4% had grades 3/4 events compared with the chemotherapy arm, wherein 34.5% of patients had grades 1/2 vomiting and 3.6% had grades 3/4 events. The incidence and severity of vomiting seems dose-dependent. Vomiting experienced by patients was proportional to the dose of claudiximab administered. Vomiting was reported in 60 to 69% of patients receiving medium and experimental doses of claudiximab compared with <40% of patients on chemotherapy alone. The investigators concluded that claudiximab in combination with first line chemotherapy provided clinically relevant benefit in PFS and OS in patients with CLDN18.2 positive gastric and GEJ adenocarcinoma (NCT01630083).

## Testing for claudin 18.2 positivity: CLAUDETECT™ 18.2

All the above studies showed benefits in the patients with tumor positive for CLDN18.2 expression of ≥2+ in ≥40% tumor cells. Of note, only 48% of the patients in the FAST study were considered positive for CLDN18.2 [28]. The biomarker’s presence in tumor specimens was determined by CLAUDETECT™ 18.2, a pathology test developed by Ganymed (now acquired by Astellas), the drug maker and sponsor of the trial. CLAUDETECT™ 18.2 is a semi-quantitative immunohistochemical assay that selectively and exclusively determines CLDN18.2 protein expression in formalin-fixed, paraffin-embedded tumor tissues from patients with adenocarcinoma of the stomach, gastroesophageal junction, and pancreas as well as bile duct and lung. CLAUDETECT™ 18.2 is the first and only CE marked in vitro diagnostic (IVD) test which allows to assess the expression levels of claudin 18.2 (CLDN18.2) in gastroesophageal, pancreatic, lung, and other solid tumors.

## Future outlook

The major limitation will be the commercial easy availability of the testing for CLDN18.2. The need for further studies revolves around finding the ideal cut-off point for the CLDN18.2 levels, which suggest the need for studies comparing outcomes between low CLDN18.2 levels vs higher levels are warranted. Another notable point in the FAST study is that the outcomes in the EOX only arm were not similar to the landmark trial REAL 2 study [OS of 11.2 (REAL 2) vs 8.7 (FAST)] [29], which could be due to patient selection. In addition, it may be interesting to know the outcomes of claudiximab-based chemotherapy compared to other regimens including Her2 directed therapy.

## Conclusions

Targeted therapy is clearly the future of gastric cancer treatments. Newer concepts must implement multi-target strategies in order to induce durable remissions. Claudiximab is a novel recombinant chimeric monoclonal antibody, which has demonstrated a significant efficacy and safety profiles when combined with other standard chemotherapy drugs and as a monotherapy in gastro-esophageal cancer in early clinical trials. It is a very promising targeted therapy for these difficult-to-treat malignancies. A phase III trial is scheduled to be started in 2017, which hopefully will provide more definitive answers and bring this agent closer to approval by the drug regulatory agencies.

## Abbreviations

ADCC: Antibody-dependent cytotoxicity
AEs: Adverse events
CDC: Complement dependent cytotoxicity
CE: Conformité Européene
CLDN: Claudin
CR: Complete response
EOX: Epirubicin, oxaliplatin, Xeloda (capecitabine)
IL2: Interleukin-2
IMAB: Ideal monoclonal antibody
ORR: Objective response rate
PFS: Progression-free survival
PR: Partial response
RR: Response rate
SD: Stable disease
ZA: Zoledronic acid
